# Clinicopathologic study of 1176 salivary gland tumors in a Chinese population: Experience of one cancer center 1997–2007

**DOI:** 10.3109/00016489.2012.662715

**Published:** 2012-04-12

**Authors:** Yu-Long Wang, Yong-Xue Zhu, Tong-Zhen Chen, Yu Wang, Guo-Hua Sun, Ling Zhang, Cai-Ping Huang, Zhuo-Ying Wang, Qiang Shen, Duan-Shu Li, Yi Wu, Qing-Hai Ji

**Affiliations:** 1 Department of Head & Neck Surgery, Cancer Hospital, Fudan University, Shanghai, China; 2 Department of Oncology, Shanghai Medical College, Fudan University, Shanghai, China; 3 Department of Pathology, Cancer Hospital, Fudan University, Shanghai, China

**Keywords:** Lymphoepithelial carcinoma, salivary duct carcinoma, incidence, fine needle aspiration, frozen section

## Abstract

*Conclusion*: Chinese patients have a higher rate of lymphoepithelial carcinoma (LEC) and salivary duct carcinoma (SDC). Comprehensive use of diagnostic modalities, neck dissection, and postoperative radiation will improve the treatment results for salivary gland tumors (SGTs). *Objectives*: To study the clinicopathological characteristics of SGTs in a Chinese population. *Methods*: The records of SGT patients operated in a tertiary cancer hospital of China were retrieved. *Results*: From December 1997 to December 2007, 289 malignant and 887 benign SGTs were operated at Cancer Hospital, Shanghai, China. Pleomorphic adenoma and Warthin's tumor were the most common types of SGT. Mucoepidermoid carcinoma (24.6% of malignant cases) and adenoid cystic carcinoma (18.0%) were the most frequent malignant cases, followed by acinic cell carcinoma (12.1%), LEC (9.7%), and SDC (9.3%). The sensitivity and specificity of ultrasound scan, fine needle aspiration biopsy, and frozen section were 58.3 and 88.6%, 87.2 and 96.7%, 86.9 and 99.6%, respectively. Neck dissections and postoperative radiation were carried out for 48.6 and 48.0% of carcinomas, respectively. The percentage of tumors by pathologic TNM stage were 23.7% for stage I, 32.9% for stage II, 17.3% for stage III, and 26.1% for stage IV. The 5-year overall survival rate was 88.0%.

## Introduction

Salivary gland tumors (SGTs) are relatively uncommon lesions, accounting for less than 3% of all head and neck tumors [1,2]. In Shanghai city, China, the standardized incidence rates of malignant neoplasm of salivary glands are 0.45 and 0.40 per 100 000 women and men, respectively. The rarity of SGT coupled with its complex and changing classification over time has made the diagnosis and management challenging.

Geographical variations in the prevalent site and type of SGTs have been reported in the literature. In the Chinese population, especially from the Chinese mainland, there have been few reports of SGTs [3–8]. Due to the higher incidence of nasopharyngeal carcinoma (NPC) and Epstein–Barr virus (EBV) infection in the Chinese population, the differential diagnosis of lymphoepithelial carcinoma (LEC) and metastatic undifferentiated carcinoma of the parotid lymph node is clinically significant for a parotid mass. These differences make a systematic audit of SGTs in mainland Chinese patients necessary and informative.

The accurate preoperative diagnosis of SGTs depends on clinical findings, imaging characteristics and, more recently, fine needle aspiration (FNA) diagnosis. FNAis a quick, minimally invasive procedure that has gained wide acceptance as a firstline diagnostic procedure for salivary gland lesions [9]. An intraoperative frozen section (FS) is obtained to confirm the diagnosis by FNA, to establish a diagnosis when FNA is nondiagnostic, and to determine the extent of tumors and assess surgical margins [10]. The accuracy of these preoperative and intraoperative diagnostic methods has been reviewed in the literature [10]. However, very few studies have discussed the comprehensive use of these methods in the management of SGTs, especially among Chinese patients with a higher incidence of NPC and EBV infection.

The objectives of this study were to determine the relative frequency and distribution of various types of SGTs in Chinese patients treated at a single cancer hospital and the clinical management and results for these patients, and to provide data for comparison with other studies in different geographic locations and/or racial populations.

## Material and methods

Medical records of consecutive patients with SGTs who underwent inpatient surgery at the Department of Head & Neck Surgery, Cancer Hospital, Fudan University, Shanghai, China from December 1997 to December 2007 were reviewed. Patient information such as age, sex, tumor characteristics, and tumor pathology were recorded in a database. Histological slides were reviewed and classified by the pathologists in the same hospital according to the World Health Organization (WHO) classification system for head and neck tumors. This research was approved by the institution's ethics committee. All the files were re-checked by the authors, and the histological slides were reviewed again by pathologists before this study. Data were analyzed using SPSS 11.0 software (SPSS, Chicago, IL, USA), and a *p* value < 0.05 was considered statistically significant.

Most of the patients underwent an ultrasound scan examination and computed tomography (CT) or magnetic resonance imaging (MRI) before surgery. An FNA biopsy was performed by specialized cytopathologists. When patients were diagnosed as having lymphoma by the cytopathologists, they had an outpatient biopsy only, with no further surgical intervention, and were thus excluded from the current study. FS was done for most of the patients during surgery. Since Chinese patients have a higher rate of NPC, an MRI and epipharyngoscope examination of the nasopharynx were done when necessary to differentiate original parotid LEC and parotid lymph node involvement from NPC. The final pathological reports were based on hematoxylin and eosin staining and immunohistochemical analysis. Malignant cases were discussed by a multidisciplinary team, which included surgeons, radiation oncologists, and medical oncologists, in order to develop a comprehensive management plan based on the pathology report and intraoperative assessment. Patient follow-up was carried out at outpatient clinics.

Superficial parotidectomy is the standard approach for the management of a parotid tumor. When malignancy was reported by intraoperative FS, the deep parotid lobe was further excised. For a submandibular gland tumor, a submandibular triangle dissection (submandibular gland excision with level IB dissection) was preferred. For clinical suspicion of lymph node involvement, a therapeutic neck dissection was performed. A selective neck dissection was used for malignant cases with clinical N0 neck, based on a pathological diagnosis by FS and individual surgeon's assessment.

## Results

### Main clinicopathologic characteristics of SGTs

From December 1997 to December 2007, 1176 cases of SGT were treated with inpatient surgery at the Department of Head & Neck Surgery, Cancer Hospital, Fudan University, which included 289 malignant cases (24.6%) and 887 benign cases (75.4%). As shown in Table I, pleomorphic adenoma (PA) and Warthin's tumor (WT) were the most frequent SGTs. Among the malignant cases, mucoepidermoid carcinoma (MEC, 24.6%) and adenoid cystic carcinoma (AdCC, 18.0%) were the most frequent, followed by acinic cell carcinoma (ACC, 12.1%), LEC (9.7%), and salivary duct carcinoma (SDC, 9.3%). These five most common pathological types accounted for 73.7% of malignant cases. AdCC was the most frequent pathologic type of malignant cases of submandibular, minor, and sublingual salivary glands, while MEC was the most frequent type of parotid carcinoma. With respect to minor SGTs, the palate was the most common site (51.4%), followed by the tongue (18.9%), buccal mucosa (13.5%), the floor of mouth (8.1%), and the larynx (5.4%). The median (SD) age at onset was 48 (15.6) years. There were 597 male cases and 579 female cases. However, the age at onset was older for men compared with women (50.10 ± 15.78 vs 45.66 ± 14.95, *p* < 0.01).

**Table I:** Pathological type and anatomic location of 1176 cases of salivary gland tumor.

	Anatomic location				
		
Pathological type	Parotid	Submandibular	Sublingual	Minor	Total
Pleomorphic adenoma (PA)	422	186	0	12	620 (52.7%)
Warthin's tumor	205	0	0	0	205 (17.4%)
Base cell adenoma (BCA)	34	1	0	0	35 (3.0%)
Myoepithelioma	9	3	0	0	12 (1.0%)
Other benign tumor	15	0	0	0	15 (1.3%)
Mucoepidermoid carcinoma (MEC)	55	6	2	8	71 (6.0%)
Adenoid cystic carcinoma (AdCC)	15	20	4	13	52 (4.4%)
Salivary duct carcinoma (SDC)	16	11	0	0	27 (2.3%)
Acinic cell carcinoma (ACC)	31	4	0	0	35 (3.0%)
Lymphoepithelial carcinoma (LEC)	22	6	0	0	28 (2.4%)
Polymorphous low-grade adenocarcinoma (PLGA)	7	0	0	2	9 (0.8%)
Adenocarcinoma NOS (AC NOS)	8	5	0	1	14 (1.2%)
Carcinoma ex PA (Ca ex PA)	8	2	0	0	10 (0.9%)
Epithelial-myoepithelial carcinoma (EMC)	3	1	0	0	4 (0.3%)
Squamous cell carcinoma	0	2	0	0	2 (0.2%)
Other malignant tumor	12	2	0	1	15 (1.3%)
Metastatic carcinoma	4	0	0	0	4 (0.3%)
Lymphoma	13	5	0	0	18 (1.5%)
Total	879 (74.7%)	254 (21.6%)	6 (0.5%)	37 (3.2%)	1176

### Different pathologic type distribution of primarily operated cases and recurrent or persistent cases

As shown in Tables II and III, of the 1176 cases, 1019 cases were primarily operated at our institution; another 103 were primarily operated at another hospital and had a second surgery because of recurrence (after more than 3 months follow-up), and 54 cases were re-operated because of persistent disease (within 3 months after the first surgery).

**Table II:** Clinicopathological characteristics of salivary gland tumors primarily operated at the Cancer Hospital, Fudan University.

	Sex			Anatomic location				
				
Histological type	Male	Female	Age, years (mean ± SD)	Parotid	Submandibular	Sublingual	Minor	Total
Pleomorphic adenoma	209 (36.9%)	357 (63.1%)	42.9 ± 14.0	377	177	0	12	566 (55.5%)
Warthin's tumor	183 (90.6%)	19 (9.4%)	61.3 ± 9.8	202	0	0	0	202 (19.8%)
Base cell adenoma	13 (37.1%)	22 (62.9%)	50.1 ± 10.8	34	1	0	0	35 (3.4%)
Myoepithelioma	5 (50.0%)	5 (50.0%)	47.9 ± 10.1	7	3	0	0	10 (1.0%)
Other benign tumor	8 (61.5%)	5 (38.5%)	47.9 ± 13.5	13	0	0	0	13 (1.3%)
Mucoepidermoid carcinoma	18 (45.0%)	22 (55.0%)	39.5 ± 15.0	29	3	2	6	40 (3.9%)
Adenoid cystic carcinoma	15 (42.9%)	20 (57.1%)	46.0 ± 12.6	13	10	2	10	35 (3.4%)
Salivary duct carcinoma	20 (83.3%)	4 (16.7%)	58.1 ± 15.6	14	10	0	0	24 (2.4%)
Acinic cell carcinoma	12 (54.5%)	10 (45.9%)	51.6 ± 17.9	18	4	0	0	22 (2.2%)
Lymphoepithelial carcinoma	7 (63.6%)	4 (36.4%)	51.8 ± 16.8	8	3	0	0	11 (1.1%)
PLGA	3 (33.3%)	6 (66.7%)	58.2 ± 11.6	7	0	0	2	9 (0.9%)
Adenocarcinoma NOS	5 (50.0%)	5 (50.0%)	58.7 ± 13.0	6	3	0	1	10 (1.0%)
Carcinoma ex PA	4 (57.1%)	3 (42.9%)	58.3 ± 10.5	5	2	0	0	7 (0.7%)
Epithelial-myoepithelial carcinoma	2 (50.0%)	2 (50.0%)	59.8 ± 15.9	3	1	0	0	4 (0.4%)
Squamous cell carcinoma	1 (50.0%)	1 (50.0%)	36.5 ± 5.0	0	2	0	0	2 (0.2%)
Other malignant tumor	7 (77.8%)	2 (22.2%)	72.3 ± 9.0	7	1	0	1	9 (0.9%)
Metastatic carcinoma	2 (100%)	0 (0.0%)	58.0 ± 11.3	2	0	0	0	2 (0.2%)
Lymphoma	5 (27.8%)	13 (72.2%)	52.8 ± 14.2	13	5	0	0	18 (1.8%)
Total	519 (50.9%)	500 (49.1%)	48.7 ± 15.5	758 (74.4%)	225 (22.1%)	4 (0.4%)	32 (3.1%)	1019

**Table III:** Clinicopathological characteristics of reoperative cases of salivary gland tumor.

	Reason for reoperation		Anatomic location				
			
Pathological type	Recurrent	Persistent	Parotid	Submandibular	Sublingual	Minor	Total
Pleomorphic adenoma	53	1	45	9	0	0	54
Warthin's tumor	3	0	3	0	0	0	3
Myoepithelioma	1	1	2	0	0	0	2
Other benign tumor	1	1	2	0	0	0	2
Mucoepidermoid carcinoma	12	19	26	3	0	2	31
Adenoid cystic carcinoma	8	9	2	10	2	3	17
Salivary duct carcinoma	0	3	2	1	0	0	3
Acinic cell carcinoma	3	10	13	0	0	0	13
Lymphoepithelial carcinoma	12	5	14	3	0	0	17
Adenocarcinoma NOS	2	2	2	2	0	0	4
Carcinoma ex PA	2	1	3	0	0	0	3
Other malignant tumor	5	1	5	1	0	0	6
Metastatic carcinoma	1	1	2	0	0	0	2
Total	103	54	121	29	2	5	157

The median age for primarily operated cases was 49 years (range 11–86), which was significantly different between benign (826 cases) and malignant tumors (193 cases) and between different malignant cases (*p* < 0.01) as shown in Table II. When the patients were stratified by age (≤50 years and > 50 years), 75% of MEC, 57.1% of AdCC, 100% of squamous cell carcinoma (SCC), and 66.1% of PA patients were under 50 years old, while 62.5% of SDC, 71.4% carcinoma ex PA (Ca ex PA), 77.8% of pleomorphic low grade adenocarcinoma (PLGA), 75.0% of EMC (epithelial-myoepithelial carcinoma), 70.0% of AC NOS (adenocarcinoma not otherwise specified), and 88.1% of WT patients were over 50 years old (*p* < 0.01). There was a predominance of men with WT, SDC, and LEC and a predominance of women with base cell adenoma, PA, PLGA, and lymphoma, as shown in Table II. Mean ages for persistent cases and recurrent cases were 43.61 and 42.83 years, respectively. As shown in Table III, PA accounted for 51.5% of recurrent cases followed by MEC (11.7%), LEC (11.7%), and AdCC (7.8%). MEC accounted for 35.2% of persistent cases followed by ACC (18.5%) and AdCC (16.7%).

### Most frequent examinations used in the diagnostic protocol

The most frequent tools used for diagnosis were ultrasound scan (83.3%) and FNA(40.1%). CT was used in 15.7% of cases and more frequently in recurrent diseases (36.9%). As shown in Table IV, among 1019 primarily operative cases, the sensitivity (for malignancy) and specificity (for the absence of malignancy) of ultrasound scan were 58.3 and 88.6%, respectively. When malignant cases were stratified by different pathological types, the highest sensitivity was for SDC (93.8%), with lower sensitivity for ACC (33.3%) and PLGA (25.0%). The sensitivity and specificity for FNA were 87.2 and 96.7%, respectively. When different pathological types were analyzed, the highest sensitivity was for SDC (100%), AC NOS (100%), and another six cases of less frequent type (100%). Sensitivity of FNA was lower for PLGA (0%) and Ca ex PA (50%).

**Table IV:** Results of ultrasound scan, fine needle aspiration, and frozen section of salivary gland tumor primarily operated at the Cancer Hospital, Fudan University.

	Ultrasound scan				Fine needle aspiration				Frozen section				
				
Pathological type	Benign	Malignant	Unclassified	Missed	Benign	Malignant	Unclassified	Missed	Benign	Malignant	Unclassified	Missed	Total
Pleomorphic adenoma	306	43	73	144	179	4	37	346	557	0	3	6	566
Warthin's tumor	109	12	39	42	76	1	5	120	198	1	2	1	202
Base cell adenoma	23	3	5	4	5	1	6	23	32	0	3	0	35
Myoepithelioma	7	0	0	3	3	2	0	5	8	2	0	0	10
Other benign tumor	7	0	2	4	0	1	2	10	11	0	2	0	13
Mucoepidermoid carcinoma	14	10	3	13	4	11	5	20	1	30	2	7	40
Adenoid cystic carcinoma	5	13	6	11	3	16	0	16	4	23	4	4	35
Salivary duct carcinoma	1	15	3	5	0	20	0	4	0	10	0	14	24
Acinic cell carcinoma	10	5	3	4	2	10	3	7	0	19	2	1	22
Lymphoepithelial carcinoma	1	3	3	4	0	6	3	2	2	3	0	6	11
PLGA	3	1	2	3	1	0	3	5	4	4	0	1	9
Adenocarcinoma NOS	4	4	1	1	0	3	0	7	0	6	0	4	10
Carcinoma ex PA	0	1	1	5	1	1	1	4	3	4	0	0	7
Epithelial-myoepithelial carcinoma	1	2	1	0	1	2	0	1	0	2	0	2	4
Squamous cell carcinoma	1	1	0	0	0	1	0	1	0	1	0	1	2
Other malignant tumor	2	5	1	1	0	6	0	3	1	5	0	3	9
Metastatic carcinoma	0	0	1	1	0	1	0	1	0	0	1	1	2
Lymphoma	3	3	8	4	0	5	2	11	3	11	3	1	18
Total	497	121	152	249	275	91	67	586	824	121	22	52	1019

### Intraoperative histological examinations of SGT by FS

FS was used for 94.9% of primarily operative cases performed at our department. The sensitivity and specificity were 86.9 and 99.6%, respectively. An attempt was made to type the SGTs based on FS. When the pathological types of tumor reported by FS were compared with the final pathological diagnosis, PA (97.1%) and WT (99.0%) had the highest concordance. For malignant cases, ACC had the highest concordance (85.7%) followed by SDC (70.0%), MEC (66.7%), and AdCC (61.3%). None of the PLGA (*n* = 9) and Ca ex PA (n = 7) cases were diagnosed correctly based on the FS.

### Treatment protocols and results

The most used surgical procedure for benign parotid and submandibular tumors was superficial parotidectomy and excision of submandibular gland. Among 173 cases of salivary gland carcinoma (Table II), all malignant cases (excluded metastatic carcinoma and lymphoma) that were primarily operated at our department, 80.9% (89/110) of parotid carcinomas had a total parotidectomy, 87.2% of submandibular carcinomas (34/39) had a level Ib dissection, 48.6% (84/173) of cases underwent lateral neck dissection, and 48% (83/173) of cases underwent postoperative radiation therapy. For parotid, submandibular, sublingual, and minor salivary gland carcinoma, the percentages of pathologic lymph node involvement (pN+) were 20.0%, 35.9%, 25.0, and 35%, respectively. The percentages of pathologic TNM stage were 23.7% for stage I, 32.9% for stage II, 17.3% for stage III, and 26.1% for stage IV. Active follow-up was completed in 98.3% of malignant cases and the median follow-up time was 30 months (range 1–140 months). The 5-year overall survival rate was 88%. The 5-year overall survival rates for stage I, stage II, stage III, and stage IV were 100, 97, 75, and 73%, respectively (*p* < 0.01).

## Discussion

This is the first study to evaluate patterns of SGTs operated in a Chinese cancer hospital during 1997–2007 according to the WHO 2005 classification that presents a detailed evaluation of > 1000 cases by pathologic classification, diagnostic methods, and treatment methods and results. Clinical epidemiological data on SGTs from various parts of the world can be helpful in better understanding the biology, clinical characteristics, and management protocols of SGT. Most of the SGTs are treated by oral and maxillofacial surgeons and head and neck surgeons in China, while none of the published literature on SGTs from China was from head neck surgeons [3,6,7]. When compared with our results from head and neck surgeons, the reported higher frequency of AdCC and PLGA in the published literature on mainland Chinese patients is due to the fact that more intraoral minor SGTs are operated by maxillo-facial surgeons than head and neck surgeons.

The most common benign and malignant SGTs in our study were the same as in the worldwide literature – PA followed by WT and MEC followed by AdCC [11,12]. New information in this study includes the observation of a higher rate of LEC (9.7%) and SDC (9.3%). Globally, SDC accounts for 0.2–5.1% of salivary gland carcinomas as reported in the literature [11–15]. As a type of undifferentiated carcinoma, LEC accounts for 0.4% of all salivary gland carcinomas in the AFIP Tumor Registry. LECs are associated with EBV infection, are histo-logically undifferentiated with NPC, and show a marked racial predilection for Alaskan natives, Southeastern Chinese, and Japanese individuals [16]. Because we do preoperative FNA for the patients at the outpatient clinic, most of the patients will undergo an outpatient biopsy instead of inpatient operation when lymphoma is reported by the cytopathologists. The present study only enrolled the patients who underwent inpatient operation, which caused the small fraction of lymphoma (1.5%) reported here.

Current clinical audit also compared the pathologic type of primarily operated, recurrent, and persistent SGTs. As the third most common salivary gland carcinoma, ACC ranks as the second most persistent carcinoma, which may be due to the higher preoperative misdiagnosis rate (with an ultrasound sensitivity of 33.3%). While FNA and FS sensitivities for ACC are 83.3% and 100%, the pathologic findings may change the operative plan for this type of carcinoma. The diagnostic benefits from FNA and FS of ACC lead us to discuss further the role of FNA and FS in the preoperative assessment of SGTs.

FNA is safe, simple, and well tolerated, and complications are rare [17]. With increased experience, if different pathologic type is stratified, such as undifferentiated carcinoma, further preoperative work-ups including nasopharyngeal examinations (MRI and epipharyngoscope) will help the final pathologic review because LEC is histologically indistinguishable from similarly named neoplasms of nasopharyngeal origin. The overall accuracy of FNA ranges from 81% to 98% [10,17,18]. The present study demonstrated good sensitivity (87.2%) and specificity (96.7%) of salivary gland FNA, similar to that previously reported in the literature. Because FNA is done by specialized cytopathologists and the diagnosis is generally known within 30 min of the procedure, repeat FNA is easily done. This makes FNA as performed at our center informative and accurate. In some cases, such as PLGA, Ca ex PA, and cystic lesions, the accuracy is lower, and the results should be confirmed with imaging studies and by FS. The reported sensitivity of FS ranges from 40% to 100% [18]. The sensitivity and specificity of FS in our reports were 86.9 and 99.6%, respectively. Beyond identifying or excluding malignancy at the time of surgery, pathologic typing of the salivary gland lesion is also important to determine the operative extent, which is better than FNA [18]. FNA and FS have complementary uses in the management of salivary gland lesions and their more frequent use will change the management of some special cases of SGTs, such as ACC.

With respect to therapeutic methods and results, postoperative radiation and neck dissection are two controversial issues. Radiotherapy is often used in an adjuvant role following definitive surgery for large tumors, high grade tumors, closed/incomplete excisions, and perineural or vascular invasion. In Italy, postoperative radiotherapy is used in 88% of cases [11]. In our series, the malignant cases were discussed by a multidisciplinary team to outline a comprehensive management plan based on the pathological report and intraoperative assessment; 48% of cases received postoperative radiation therapy. Neck dissection was performed for 48.6% of cases at our department, and 20% of cases were pathologic pN+. The 5-year overall survival (88%) of this series of patients exceeds treatment results reported elsewhere in the literature (46–81%) [19]. The better treatment results may be due to the good diagnostic accuracy of FNA and FS in our hands, which helps us with treatment planning. Neck dissections and postoperative radiation for elective patients can also improve the treatment results. If the accuracy of preoperative or intraoperative grade diagnosis of SGTs is low, elective neck dissection has been recommended for all the parotid cancer patients [20].

## Conclusion

This investigation is the first to describe the clinicopathological data of SGTs treated by head and neck surgeons with a larger numbers of cases during a recent 10-year period. We found that Chinese patients have a higher rate of LEC and SDC. Comprehensive use of imaging techniques, FNA, and FS will improve the diagnostic accuracy. Neck dissection and postoperative radiation for selected patients can further improve the treatment results for salivary gland carcinoma.
